# Structural Basis for the Recognition of Human Cytomegalovirus Glycoprotein B by a Neutralizing Human Antibody

**DOI:** 10.1371/journal.ppat.1004377

**Published:** 2014-10-09

**Authors:** Nadja Spindler, Uschi Diestel, Joachim D. Stump, Anna-Katharina Wiegers, Thomas H. Winkler, Heinrich Sticht, Michael Mach, Yves A. Muller

**Affiliations:** 1 Institut für Klinische und Molekulare Virologie, Friedrich-Alexander Universität Erlangen-Nürnberg, Erlangen, Germany; 2 Lehrstuhl für Biotechnik, Friedrich-Alexander Universität Erlangen-Nürnberg, Erlangen, Germany; 3 Institut für Biochemie, Friedrich-Alexander Universität Erlangen-Nürnberg, Erlangen, Germany; 4 Nikolaus-Fiebiger-Zentrum für Molekulare Medizin, Friedrich-Alexander Universität Erlangen-Nürnberg, Erlangen, Germany; University of Tübingen, Germany

## Abstract

Human cytomegalovirus (HCMV) infections are life-threating to people with a compromised or immature immune system. Upon adhesion, fusion of the virus envelope with the host cell is initiated. In this step, the viral glycoprotein gB is considered to represent the major fusogen. Here, we present for the first time structural data on the binding of an anti-herpes virus antibody and describe the atomic interactions between the antigenic domain Dom-II of HCMV gB and the Fab fragment of the human antibody SM5-1. The crystal structure shows that SM5-1 binds Dom-II almost exclusively *via* only two CDRs, namely light chain CDR L1 and a 22-residue-long heavy chain CDR H3. Two contiguous segments of Dom-II are targeted by SM5-1, and the combining site includes a hydrophobic pocket on the Dom-II surface that is only partially filled by CDR H3 residues. SM5-1 belongs to a series of sequence-homologous anti-HCMV gB monoclonal antibodies that were isolated from the same donor at a single time point and that represent different maturation states. Analysis of amino acid substitutions in these antibodies in combination with molecular dynamics simulations show that key contributors to the picomolar affinity of SM5-1 do not directly interact with the antigen but significantly reduce the flexibility of CDR H3 in the bound and unbound state of SM5-1 through intramolecular side chain interactions. Thus, these residues most likely alleviate unfavorable binding entropies associated with extra-long CDR H3s, and this might represent a common strategy during antibody maturation. Models of entire HCMV gB in different conformational states hint that SM5-1 neutralizes HCMV either by blocking the pre- to postfusion transition of gB or by precluding the interaction with additional effectors such as the gH/gL complex.

## Author Summary

Human cytomegalovirus (HCMV) belongs to the family of β-herpes viruses. HCMV infections are not only life threatening to people with a compromised immune system but also the most common viral cause of congenital defects in newborns. Hence, the development of HCMV vaccines was ranked top priority by the US Institute of Medicine in 1999. Virtually all infected individuals develop antibodies against the envelope protein gB, which plays a crucial role in the infection process. Here, we describe the crystal structure of a fragment of the virus neutralizing antibody SM5-1 in complex with an antigenic determinant of gB, namely Dom-II. The structure shows that antigen antibody interactions are concentrated within two CDRs of SM5-1. Computational methods and an analysis of additional antibody sequences from the same lineage reveal that additional key contributions to high affinity binding are provided by residues that stiffen the extra-long CDR H3 loop without directly contacting the antigen. We suggest that the optimization of such indirect contributions represents a common and yet undervalued principle of the antibody maturation process. Furthermore our data suggest that the neutralizing effect of SM5-1 either originates from blocking membrane fusion or from preventing interaction of gB with other envelope proteins.

## Introduction

Human cytomegalovirus (HCMV) belongs to the family of β-herpes viruses and is a clinically important pathogen. While infection in hosts with a functional immune system is usually clinically asymptomatic, the virus can cause significant morbidity and mortality in individuals with an immature or suppressed immune system. As such, the virus still represents a potentially severe clinical complication in transplant recipients [1]. Congenital HCMV infection is also the most common infectious cause of neurological disorders in children [2]. Hence, the development of vaccines against HCMV is considered a top priority [3].

Herpes viruses enter cells via a cascade of molecular interactions, which ultimately results in the fusion of the viral envelope with target cell membranes. In an initial step the virus attaches to the target cell surface via a non-specific, low-avidity binding to heparan sulfate proteoglycans and in subsequent steps interacts with more specific, higher avidity receptors (for review see [4]). While for some herpes viruses cellular receptors and their viral ligands have been well characterized, the situation is less clear for HCMV. On the host side, different molecules such as integrins [5], EGFR [6] or PDGF-α receptor [7] have been postulated as specific receptors. The viral ligand that was described in these studies for HCMV was in all cases glycoprotein B (gB). However, some of these findings were also challenged [8], [9].

Receptor binding initiates a cascade of events that enables fusion of viral and cellular membranes. The core fusion complex for herpes viruses in general consists of gB and gH/gL (reviewed in [10]). In the case of HCMV, gH/gL associated proteins such as gO or the UL128-131 complex determine cell tropism and/or mode of entry [11], [12]. Fusion takes place at the plasma membrane in the case of fibroblasts [13] whereas endo-/epithelial cells are infected by fusion in an endocytic compartment [14], [15], indicating that the fusion complex is functional in different pH-environments. Events similar to macropinocytosis may also be involved in HCMV infection of certain cell types, highlighting that the overall situation is relatively complex [8], [16].

It is current consensus that within the core fusion complex, gB represents the actual fusogen while gH/gL function as accessory proteins, which activate gB. Crystal structures of gB from herpes simplex virus type 1 (HSV-1) and Epstein-Barr virus (EBV) revealed that gB shares structural similarities with other type III fusion proteins from unrelated viruses such as VSV-G and baculovirus gp64 [17]–[21]. These data suggested that currently available gB structures represent the postfusion form rather than prefusion form of the proteins, although this is not proven. In analogy to VSV-G, where structures of the post- and prefusion forms are available, gB is expected to undergo a conformational transition from its original prefusion form as present in the viral envelope to the final postfusion form occurring after fusion of the viral and host cell membranes [19], [20].

The central role of gB during the fusion event makes gB a prime target for host defense mechanisms. Indeed, virtually all HCMV infected individuals develop antibodies against gB, and the neutralizing capacity of sera from HCMV convalescent individuals correlates with the anti-gB antibody titer [22], [23]. Due to its high immunogenicity, HCMV gB was selected as vaccine antigen, and phase II clinical trials using a recombinantly produced gB protein have been conducted. Partial protection was seen both in seronegative women or kidney transplant recipients [24], [25].

We recently isolated a panel of human monoclonal antibodies (mAb) against gB from healthy HCMV seropositive donors that target two novel antigenic domains (AD) in gB, termed AD-4 and AD-5 [26]. Several antibodies directed either against AD-4 or AD-5 showed neutralizing activity in nanomolar concentrations in *in vitro* assays [26]. These antibodies were shown to be effective in a postadsorption step of infection, to neutralize different virus strains and, importantly, to block infection of different target cells types such as fibroblasts, epithelial cells and dendritic cells with similar efficiency [26]. Thus, these antibodies can be considered broadly neutralizing. SM5-1, directed against AD-4, was identified as the most potent neutralizing mAB in this panel. AD-4 structurally corresponds to domain II (Dom-II) of the crystal structure of the homologous gB protein from HSV and EBV [17], [18]. The fold of Dom-II resembles that of PH domains, and in all crystal structures of type III fusion proteins, Dom-II is formed by a discontinuous protein sequence and comprises in strain HCMV AD169 amino acids 121–132 and 344–438 of gB [26], [27]. Interestingly, in case of HSV it was shown that antibody binding to Dom-II not only neutralizes HSV but at the same time blocks interaction with gH/gL [28]. Since the sequence of Dom-II is highly conserved between different HCMV isolates and since interaction of gB with gH/gL is a prerequisite for activation of the fusion machinery in all herpes viruses blocking this interaction by molecular effectors appears to be a promising strategy for preventing infections.

Here, we report on the first crystal structure of a complex between a neutralizing antibody and a herpes virus envelope protein domain, namely the Fab fragment of the AD-4 -specific monoclonal antibody SM5-1 in complex with an isolated HCMV gB Dom-II. The structure shows that the molecular determinants for picomolar binding cluster within light chain complementarity determining region 1 (CDR L1) and predominantly within an unusually long heavy chain CDR H3 of the antibody. An alignment of additional antibody clones from the same germline lineage in combination with structural analysis and molecular dynamics (MD) calculations highlighted important structural aspects of the somatic maturation process and the concomitant emergence of high affinity antibodies. We expect the results of our study to inform the structure-based design of anti-HCMV vaccines.

## Results

### Crystal structures of HCMV gB Dom-II in complex with SM5-1 Fab and of the isolated proteins

**Figure 1:**
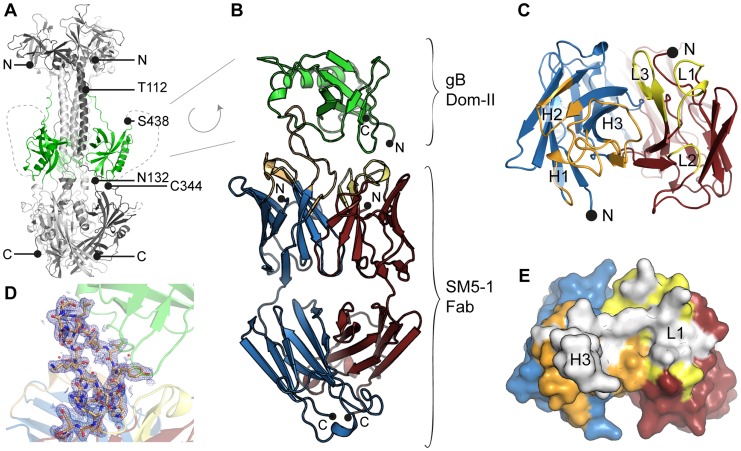
HCMV gB Dom-II recognition by antibody SM5-1. (A) Trimeric model of HCMV glycoprotein B (gB) derived from the crystal structure of the homologous HSV-1 gB protein (PDB ID 2gum, [18]). Of the different gB domains, Dom-II is highlighted in green. N- (Thr-112) and C-terminal (Ser-438) amino acids of the Dom-II expression construct are labelled. Dashed lines represent regions that were not resolved in the crystal structure of HSV-1 gB and are therefore excluded from the model. (B) Cartoon representation of the Dom-II-SM5-1 Fab complex determined at an atomic resolution of 2.1 Å. Dom-II is colored in green, SM5-1 light and heavy chains are colored in red and blue, respectively. The CDR loops of the heavy chain are shown in orange and those of the light chain in yellow. Two segments of gB (residues 112–132 and 344–438) were fused together by an artificial linker segment in order to obtain a sequence-contiguous Dom-II protein [32]. (C) Top view of the antigen-binding site with the structure of Dom-II omitted in the presentation. Colors are as in (B). CDR loops are labeled L1 to L3 and H1 to H3. (D) 2mFo-DFc map contoured at 1σ and displayed within 2.5 Å around any CDR H3 atoms. Contiguous electron density is observed for the entire H3 loop in the antigen-bound structure. (E) Surface representation of the antigen-binding site of SM5-1 oriented as in (C). Shown in white is the surface area of SM5-1 that becomes buried upon antigen binding.

**Figure 2:**
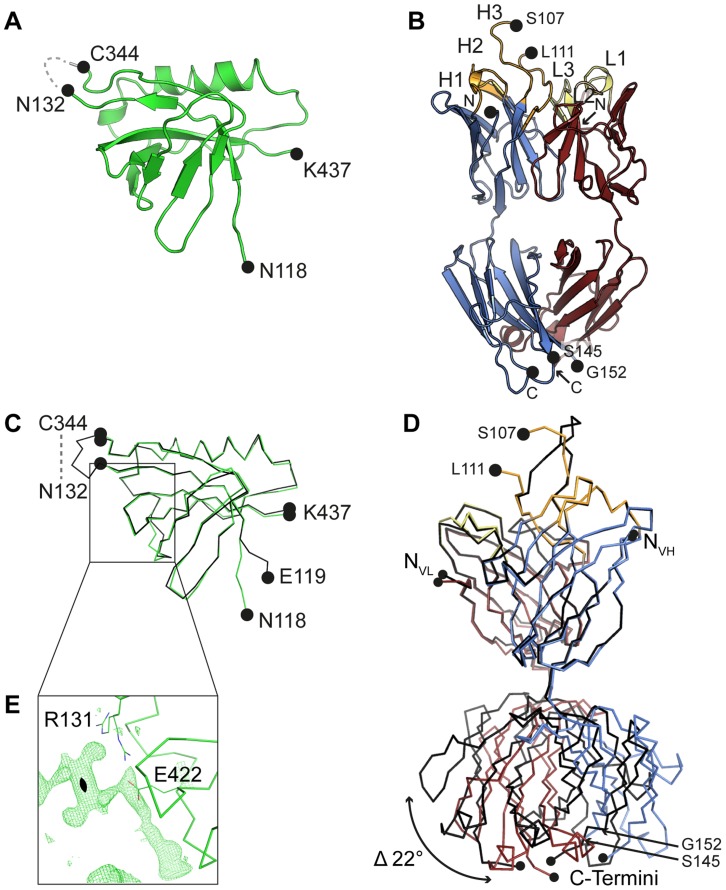
Crystal structures of unbound Dom-II and SM5-1. (A) Cartoon representation of unbound engineered Dom-II solved at 1.8 Å resolution. No electron density is observed for the artificially introduced linker amino acids (grey dashed line). (B) Cartoon representation of unbound Fab SM5-1 solved at 1.9 Å resolution. The heavy and light chains are colored in blue and red, respectively. The CDR loops are depicted either in orange (H1 to H3) or yellow (L1 to L3). In the free SM5-1 structure, the polypeptide chain between residues H3 107 and 111 as well as heavy chain residues 145 to 152 could not be traced. (C) Superposition of Dom-II in the unbound (in green) and Fab SM5-1-bound state (in black). (D) Superposition of Fab SM5-1 in the unbound state (in color) and in the Dom-II-bound state (in black). The variable domains were superimposed, and the superposition shows that the differences between both structures are limited to CDR H3 and changes in the elbow angle (indicated by an arrow). (E) Close-up showing residual positive mFo-DFc difference electron density in the unbound Dom-II crystal structure. The density is contoured at a 3σ level and extends across a crystallographic two-fold axis (black oval).

The crystal structure of an engineered variant of Dom-II from HCMV gB was solved in complex with the antigen-binding fragment (Fab) of antibody SM5-1 at 2.1 Å resolution (Table 1, Fig. 1). In addition the structures of engineered Dom-II and of the SM5-1 Fab fragment were solved individually at resolutions of 1.8 and 1.9 Å, respectively (Table 1, Fig. 2). In the engineered Dom-II fragment, residues 112–132 and 344–438 of HCMV gB were linked by an artificial 5-residue-long linker segment in order to produce a sequence-contiguous Dom-II domain (Fig. 2) [26]. Although no electron density is visible for the linker residues and a few N- and C-terminal residues in the structure of unbound Dom-II, the overall structure compares well with the structures of discontinuous Dom-IIs from full-length gB proteins [18]. When considering all common main chain atoms, the structure of engineered unbound HCMV Dom-II differs from that of Dom-II from HSV-1 gB (PDB entry: 2gum [18]) by an r. m. s. deviation of 1.7 Å and from Dom-II from EBV gB (PDB entry: 3fvc [17]) by a deviation of 1.5 Å. These deviations closely match expectations from sequence differences. Whereas the overall sequence identity between HCMV and HSV-1 gB is 28% (UniProt sequences P06437 vs P06473 [29]), the identity between the corresponding Dom-II segments is 34% (HCMV gB residues 119–132 and 344–438 *versus* HSV-1 gB residues 142–153 and 364–459). HCMV gB Dom-II aligns significantly worse with analogous VSV-G Dom-III in either the pre- or postfusion form (PDB entries 2j6j and 2cmz [19], [20]). Here, the r. m. s. deviations range from 3.1 to 3.3 Å.

**Table 1:** Crystallographic data collection and refinement statistics.

Data statistics	gB Dom-II	SM5-1 Fab	Dom-II-SM5-1 Fab complex
Wavelength (Å)	0.91841	0.91841	0.91841
X-ray source, detector	Bessy BL14.1, Rayonics MX-225 3×3 CCD	Bessy BL14.1, Rayonics MX-225 3×3 CCD	Bessy BL14.1, Rayonics MX-225 3×3 CCD
Space group	P6_1_22	P4_1_2_1_2	P2_1_
Unit cell parameters	a = b = 89.9 Å, c = 75.7 Å, α = β = 90°, γ = 120°	a = b = 92.6 Å,c = 124.6 Å, α = β = γ = 90°	a = 43.1 Å, b = 69.4 Å, c = 101.0 Å, α = γ = 90°, β = 99.7°
Matthews Coefficient (Å^3^/Da^−1^)	3.04	2.55	2.35
No. molecules/ASU	1	1	1
Solvent content (%)	59	51	47
Resolution (Å)+	1.76 (1.81–1.76)	1.87 (1.98–1.87)	2.11 (2.16–2.11)
No. of reflections (Unique)	18,070 (1,297)	45,558 (7,187)	33,553 (2,475)
Redundancy+	10.7 (11.1)	8.1 (8.2)	3.2 (3.2)
Completeness (%)+	98.1 (97.2)	99.9 (99.2)	98.5 (98.6)
Mean *I*/(σ*I*)+	26.04 (3.1)	27.8 (5.8)	12.4 (2.3)
*R*_sym_ (%)+	5.3 (93.5)	5.9 (40.2)	7.7 (65.9)
Wilson B-factor (Å^2^)	34.3	30.3	39.9
**Refinement statistics**			
**Final ** ***R*** **-factor (%)**			
Working set	20.18	18.60	18.02
Working set+test set	20.31	18.83	18.31
Free *R*-factor (%)#	22.82	23.15	23.76
**R. m. s. deviations**			
Bond lengths (Å)	0.010	0.019	0.017
Bond angles (°)	1.387	2.034	1.86
**Mean B-value (Å^2^)**			
Protein atoms	36.3	30.3 (heavy chain), 29.0 (light chain)	38.1 (Dom-II), 37.0 (heavy chain), 39.0 (light chain)
Solvent/additional molecules	41.5 (solvent molecules)	33.3 (solvent), 39.8 (formate), 29.4 (Na^+^ atoms)	39.9 (solvent), 66.5 (Hepes molecule), 39.2 (ethylene glycol)
**Anisotropic overall scaling factors (Å^2^)**			
B11, B22, B33	0.33, 0.33, −1.08	−0.10, −0.10, 0.21	0.76, −0.87, 0.07
B12, B13, B23	0.33, 0.0, 0.0	0.0, −0.0, 0.0	−0.0, 0.22, −0.0
No. of protein atoms	963	1723 (heavy chain), 1619 (light chain)	904 (Dom-II), 1738 (heavy chain), 1591 (light chain)
No. of additional molecules/atoms	76	386 (solvent), 7 (formate molecules), 2 (Na^+^ atoms)	243 (solvent), 1 (HEPES molecule), 3 (ethylene glycol molecules)
**Ramachandran plot (%)** ##			
preferred regions/allowed/outlier	98.9/1.1/0.0	96.3/3.2/0.5	95.4/3.9/0.7

+ Numbers in parentheses are for the highest-resolution shell.

# 5% of reflections have been chosen as *R*_free_ set.

## Calculated with program COOT [67].

HCMV gB Dom-II folds into an all-antiparallel β-sheet sandwich and consists of a 4-stranded and a 5-stranded β-sheet that are oriented at almost a right angle to each other. A five-turn-long α-helix interconnects two of the β-strands in the 5-stranded β-sheet. As has been noted before the domain topology of Dom-II resembles that of pleckstrin homology (PH) domains with the caveat that the mentioned α-helix is absent in PH domains [18], [30]. Moreover, PH domains display an alternative α-helix, namely after the last β-strand. This helix is missing in EBV gB and in a previous HSV-1 gB structure (PDB entry 2gum) [17], [18]. However, such a PH domain-like helical segment extends from Dom-II in a more recent crystal structure of HSV-1 gB (residues 463–475, PDB entry: 3nwf) [31]. It should be noted that this helical extension is not present in the HCMV gB Dom-II structure reported here, since the corresponding residues (442–454) were not included in engineered Dom-II [32]. Also, HCMV gB, in contrast to HSV-1 gB, is posttranslationally cleaved after position 459 and this might influence the local structure [33], [34].

The closest structurally related PH domain that can be identified by a DALI search is that of the Rho GTPase-activating protein 27 (ArhGAP27, PDB entry 3pp2). The r. m. s. deviation is as high as 3.0 Å when calculated for 75 out of 115 possible equivalent Cα positions [35]. Until now, no indications exist that Dom-IIs in herpes viruses use any of the canonical ligand interactions sites observed in PH domains for binding either phosphotyrosines, polyproline helices or inositol phosphate head groups [30]. To our knowledge, this also extends to protein-protein interaction modes [30]. Interestingly, with respect to the possible involvement of Dom-II in protein ligand interactions, we observe extensive residual positive difference electron density along a face of Dom-II of HCMV gB, namely close to Arg131 and Glu422 (Fig. 2E). Although the identity of the ligand cannot be inferred from the composition of the crystallization solution, the elongated shape of the electron density suggests that the ligand might correspond to a peptide. The ligand straddles a hydrophobic pocket in Dom-II and surprisingly the same pocket is also targeted by CDR H3 of the antibody SM5-1 in the structure of the antibody antigen complex (see below).

The structure of unbound SM5-1 resembles that of other Fab fragments. No significant conformational changes occur in either SM5-1 or Dom-II upon complex formation with exception of the conformation of CDR H3 of SM5-1 and changes in the elbow angle (Fig. 2D). In the complex, CDR H3 participates in extensive contacts with Dom-II, and all residues within CDR H3 become clearly defined in the electron density map (Fig. 1D). Overall, the r. m. s. deviations between the free *versus* bound structures of Dom-II and SM5-1 are very low. They range from 0.27 Å for the V_H_-V_L_ domain pair of SM5-1 to 0.33 Å for Dom-II and 0.72 Å for the C_H1_-C_L_ domain pair of SM5-1. Upon formation of the complex, the elbow angle between the variable and constant domains of SM5-1 changes from 170° in the free state to 195° in the bound state (Fig. 2D) [36].

### Molecular determinants of antigen recognition

**Figure 3:**
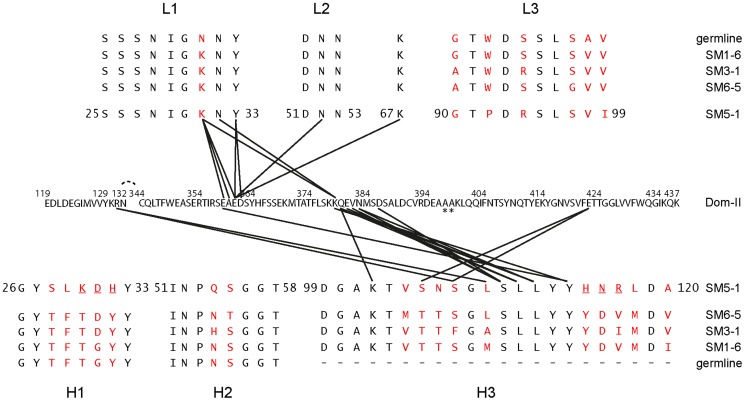
Contacts between Dom-II and SM5-1 together with an alignment of the CDRs of related antibodies. Black lines indicate intermolecular distances smaller than 3.5 Å. The Dom-II sequence is reported in its entirety, while for SM5-1 only the amino acid sequences of the CDRs and of frame work residue Lys67 are displayed. Asterisks indicate mutations in engineered Dom-II in the complex. The CDR sequences of the highly related antibodies are provided and listed either above or below the SM5-1 sequence. Amino acids that differ in any of the sequences are highlighted in red. SM5-1 residues that were substituted *in silico* for the molecular dynamics simulations are underlined.

**Figure 4:**
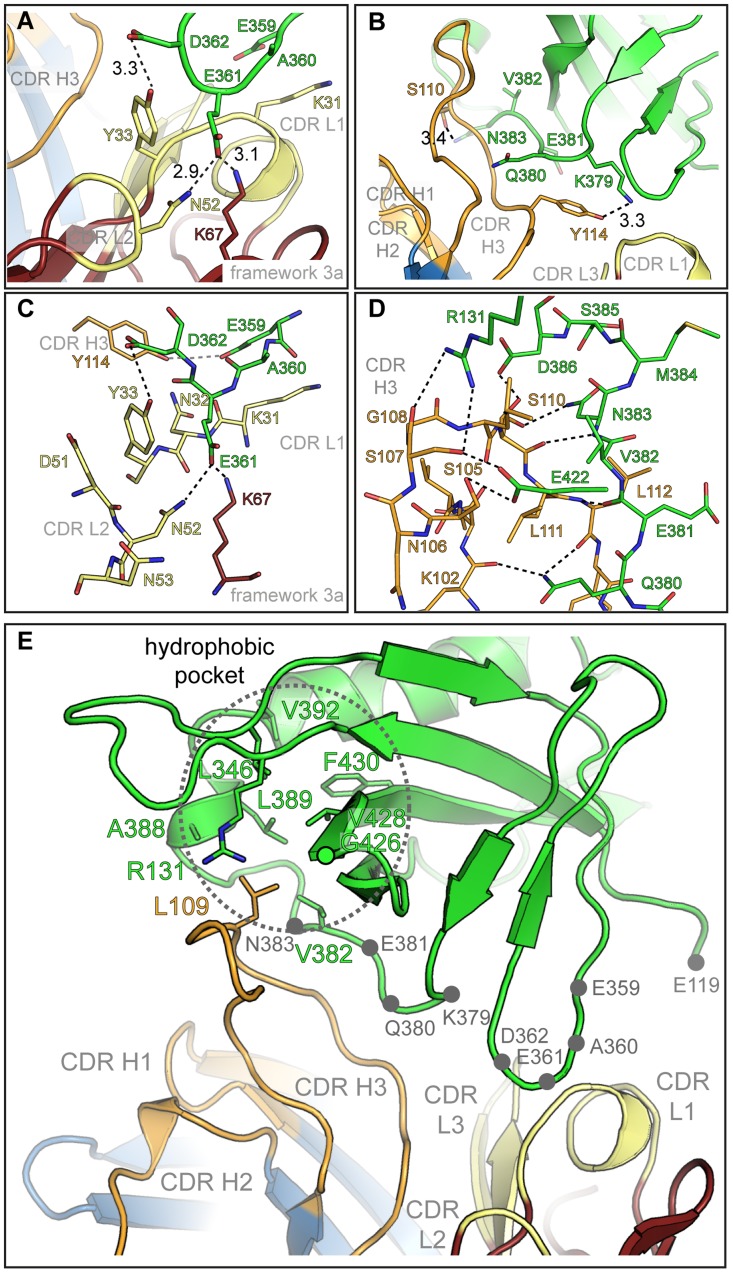
Details of the Dom-II-SM5-1 Fab recombining site. (A) Side chain-specific interactions between CDR H3 and residues 359-EAED-362 of Dom-II. Hydrogen bond distances are reported in Å. (B) Focus on the interaction between SM5-1 and Dom-II residues 379-KQEVN-383. (C, D) Alternative representation of the interactions formed between SM5-1 Fab and Dom-II segment 359-EAED-362 (C) and 379-KQEVN-383 (D). Hydrogen bonds within a distance of <3.5 Å are depicted in black, >3.5 Å in grey. (E) Residue Leu109 from CDR H3 shields a hydrophobic pocket in Dom-II. Side chains lining the hydrophobic cavity (dotted circle) are shown as sticks models. Grey dots highlight the segments 359-EAED-362 and 379-KQEVN-383 of Dom-II.

Inspection of the antigen-antibody-combining site shows that HCMV gB Dom-II recognition is achieved predominantly *via* an unusually long heavy chain CDR H3 with additional contributions from CDR L1 (Fig. 1E, Fig. 3). Of the total solvent accessible surface area of 775 Å^2^ buried by SM5-1 in the complex interface, H3 contributes 515 Å^2^ (66.5%) and L1 210 Å^2^ (27%) (Fig. S1). Although changes in surface accessibility are observed for residues in additional CDRs upon binding, no residue in CDR L3, H1 and H2 makes direct contacts with Dom-II as judged by an inter-atom distance cutoff >3.5 Å, and CDR L2 contributes only a single hydrogen bond to the (Fig. 3, 4A and C).

In antibody-antigen structures CDR H3 donates almost always the most extensive surface area contribution to the antigen-combining site, however, a scenario where antigen-binding is achieved almost exclusively by only two out of six CDRs remains unusual [37]. Antigen recognition in SM5-1 is reminiscent of the recognition of the class I fusion protein haemagglutinin from influenza A by the C05-Fab fragment [38]. Here also, a 24-residue-long H3 loop extends from the surface of the antibody and, in combination with H1, suffices for antigen recognition. In case of SM5-1, antigen recognition also includes a contribution from framework residue 3A, namely Lys67 of the light chain (Fig. 3).

The residues in Dom-II that are recognized by SM5-1 cluster preferentially within two contiguous peptide segments, namely 359-EAED-362 and 379-KQEVN-383 (Fig. 3 and 4). Whereas 359-EAED-362 interacts mainly with light chain CDR L1, 379-KQEVN-383 participates in a broad and extended hydrogen-bonding network with CDR L1 and preferentially with CDR H3 (Fig. 4A–D). SM5-1 recognizes in addition a highly hydrophobic pocket on the surface of Dom-II. This pocket is located at the same position where we observed residual positive difference electron density in the structure of unbound Dom-II (Fig. 2E). The CDR H3 segment 105-SNSGLSLL-112 binds across the opening of this pocket, and the side chain of Leu109 points into the pocket (Fig. 4E). However, Leu109 appears not to be able to completely fill out this pocket (Fig. S2A). The Leu109 contact is sealed-off from the surrounding solvent by a number of hydrogen bond interactions and these mainly involve the backbone atoms from the adjacent serine and leucine residues in CDR H3. These hydrophilic contacts also include a bidental interaction between Dom-II residue Arg131 and CDR H3 residue Ser107 that appears to stabilize the conformation of CDR H3 (Fig. 4D). Overall, the atomic architecture of the interaction patch that contains CDR H3 residue Leu109 resembles that typically observed for protein interaction hot spots [39].

Residue Tyr364 and the segment 378-KKQE-381 were identified as key determinants for binding and neutralizing activity of a series of SM5-1 related antibodies in a previous mutagenesis study [32]. The two residues Tyr364 and Lys379 were especially critical for SM5-1 binding (YK-motif, [32]). In the structure of the complex, Lys379 adopts a central role, since, together with Glu359, it is the only Dom-II residue that contacts both CDRs L1 and H3 (Fig. 3, Fig. S2B). In contrast, Tyr364 does not directly bind to SM5-1. It lays in immediate spatial proximity to Dom-II residues Glu359 and Lys379 and shields the side chain of Lys379 from the surrounding water molecule. Its role might be to help orienting key Dom-II residues that directly interact with SM5-1 (Fig. S2B). Overall, the structural data presented here are in good agreement with the previously reported mutational analysis [32].

### Structural determinants of the somatic selection and maturation process

SM5-1 belongs to a series of anti-HCMV gB mAbs that were isolated from an individual donor at a single time point [26]. All clones are related, i.e. are derived from one B-cell that was clonally diversified by somatic mutation and selection during germinal center reaction. Since all residues that interact with the antigen in the structure of the complex are largely conserved in these clones it seems reasonable to assume that the structural mode of Dom-II-recognition is conserved among these antibodies (Fig. 3). The binding affinities of the individual antibodies against HCMV gB differ by more than two orders of magnitude, and the highest affinity is observed for SM5-1 (Table S1) [26], [32]. The latter observation is consistent with the fact that SM5-1 is the most mature antibody of this family since it displays the largest number of mutations compared to the germline sequence (Fig. 3). When comparing the dissociation constants to the neutralization activities of the related mAbs, a clear correlation can be observed. However, the neutralization activities do not increase by the same extent as the binding affinities suggesting that affinity maturation as it occurs in the germinal center reaction does not translate directly into a better neutralization activity against HCMV. Interestingly, the off-rates of the antibodies (i.e. the dissociation rate) correlate best with their neutralization activities.

**Figure 5:**
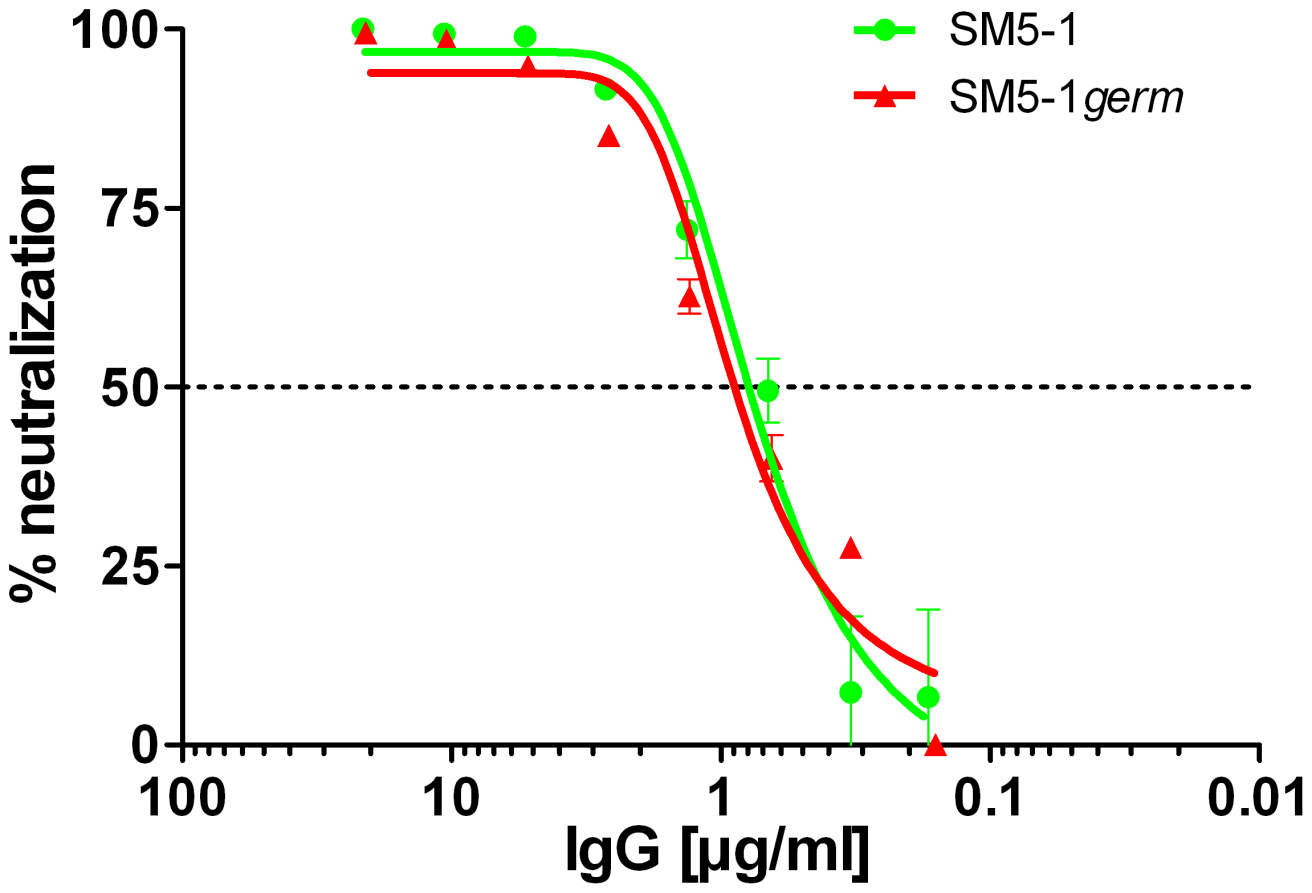
Neutralization capacity of SM5-1 and of partially germline-reverted SM5-1*germ*. Viruses were preincubated with the antibody for 1 h and fibroblasts were infected with the virus-antibody mixture. Medium was changed 4 h after infection. 24 h post infection luciferase activity was measured in relative light units (RLU) as described previously and percent neutralization was calculated [26]. Shown is one data set from two independent experiments.

In SM5-1 the CDRs L1 and H3 but also CDRs L3, H1 and H2 clearly underwent affinity maturation from the germline sequence (Fig. 3). However in the crystal structure the latter three CDRs do not interact with Dom-II. This raises the possibility that these CDRs contribute to the enhanced neutralization activity of SM5-1 and interact with parts of gB or even additional viral components that are not present in recombinant Dom-II. To test this hypothesis SM5-1*germ* was generated, a SM5-1 derivative in which the CDRs H1, H2 and L3 were reverted to the respective germline sequence (Fig. S3). In neutralization assays we could not detect any difference between SM5-1 and SM5-1*germ*, indicating that affinity maturation within those CDRs which are not in contact with recombinant Dom-II has no measurable impact on the neutralization activity of SM5-1 (Fig. 5). These data further strengthen the notion that the neutralization capacity of SM5-1 rests entirely on the interaction mode between DomII and SM5-1 that has been mapped in the crystal.

The data above also suggests that affinity-maturated residues of SM5-1, which do not contact Dom-II, might rather play a role for stabilizing the structure of the antibody itself. Inspection of the SM5-1 structure reveals that indeed several of the respective residues form tight intramolecular interactions including Lys30, Asp31, and His32 of CDR H1. As an example, His32 from CDR H1 contacts Asp99 located at the N-terminus of the long CDR H3. This region is further stabilized by additional polar interactions of residues His115, Asn116, Arg117 of CDR H3. The latter residues have emerged during affinity maturation of SM5-1 and also do not form direct interactions with the antigen. To investigate the role of these polar residues in more detail, molecular dynamics (MD) simulations were performed for SM5-1 and for a 6-fold *in silico* substituted variant SM5-1*, in which the respective residues of CDR H1 and H3 were replaced to match the sequence of the less mature SM1-6 that binds Dom-II with lower affinity (heavy chain K30T, D31G, H32T, H115Y, N116D and R117V).

**Figure 6:**
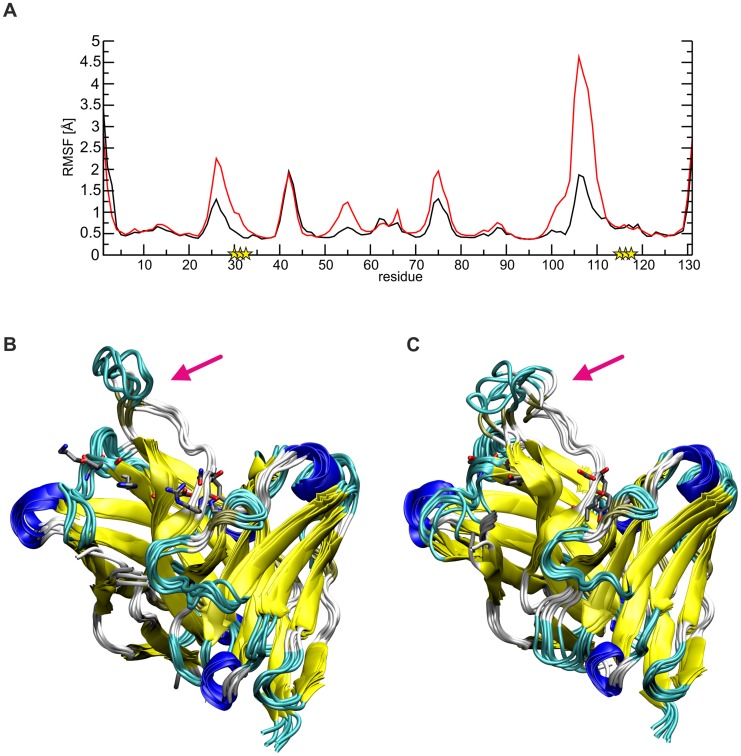
Conformational flexibility of SM5-1 and SM5-1*. (A) Plot of the root mean square fluctuations (RMSF) per residue indicating the enhanced flexibility of residues 102–112 (CDR H3) in SM5-1* (red line) compared to SM5-1 (black line). Sequence positions that differ between SM5-1 and SM5-1* are marked by a yellow asterisk. (B, C) Overlay of 6 structures collected every 20 ns over the simulation time for SM5-1 (B) and SM5-1* (C). Note that the CDR H3 loop in SM5-1* exhibits a higher flexibility and deviates further from the Dom-II-bound conformation which served as starting structure. The six residues that are different between SM5-1 and SM5-1* are shown in stick presentation and a pink arrow points towards the CDR H3 loop.

Comparison of the dynamics of SM5-1 and SM5-1* reveals that the substitutions mainly enhance the flexibility of the long CDR H3 loop, and this is most pronounced for residues 104–113 (Fig. 6). The overlay of the structures collected over the simulation time also showed a more prominent deviation of CDR H3 of SM5-1* from the Dom-II-bound conformation, which served as starting structure for the simulation (Fig. 6B,C).

The higher flexibility of CDR H3 in SM5-1* can be attributed to the loss of important stabilizing side chain interactions in the anchor region of CDR H3. For example, in SM5-1 Asp99 is fixed by multiple H-bonds with the side chain of His32, Tyr113 and His115 as well as with the backbone of Tyr33 and Asn116 (Fig. S4). Due to the replacement of His32 and His115 with tyrosine, this compact network of interactions involving the N- and C-terminus of CDR H3 is lost leading to a destabilization of the long connecting loop. The same effect is also detected in a control MD simulation starting from the structure of unbound SM5-1 (Fig. S5). Thus, it appears that the specific role of a number of affinity matured residues in SM5-1 is not to directly interact with Dom-II but to stabilize the binding-competent conformation of CDR H3.

## Discussion

### Affinity maturation of SM5-1 involves intramolecular stabilization of the long CDR H3 loop

Analysis of the structure of SM5-1 in complex with gB Dom-II revealed that a significant portion of the mutated residues do not directly interact with Dom-II. MD simulations provide a functional explanation for this puzzling finding and suggest that these residues play a role in the stabilization of the SM5-1 CDR H3 loop itself. Intramolecular CDR H3 stabilization of antibodies has been reported previously and can be achieved by the formation of hydrogen-bonded β-hairpins, disulfide bridges, or the enrichment of prolines [40], [41]. All these structural features are expected to enhance rigidity, which will reduce the entropy changes upon antigen recognition and, therefore, result in higher binding affinity.

Long CDR H3s frequently exhibit extended β-hairpin structures as exemplified by the HIV-1 gp120-binding antibodies 10-1074 or PGT122 [42], [43]. The CDR H3 of SM5-1 adopts an extended conformation, but does not display a regular β-hairpin structure. Consequently, the ability to gain intramolecular stabilization by backbone hydrogen bonds is rather limited; however, this is compensated by an additional stabilization through side chain hydrogen bonds. A similar combination of backbone and side chain hydrogen bonds is also present in the long CDR H3 of the haemagglutinin-binding human mAB C05, which resembles a hammerhead topology [38]. As is the case for SM5-1, the CDR H3 conformation of C05 does not significantly change upon antigen binding suggesting that intramolecular stabilization of long CDR H3 loops favors antigen binding by reducing the entropic loss caused by the interaction [38].

In contrast to backbone hydrogen bonds, which can be formed by any amino acid, the formation of side chain hydrogen bonds requires the presence of distinct amino acids with functional side chain groups. Thus, the emergence of polar residues during antibody maturation may reflect an enhanced intramolecular stabilization and does not necessarily imply that the respective residue forms interactions with the antigen.

Consequently, this observation has also implications for other areas of research: Firstly, it underscores the difficulty of predicting correct antibody-antigen structures by docking approaches. These approaches frequently consider all residues, which have emerged during affinity maturation, as part of the interface. Such an approach will consequently fail in cases like SM5-1 by producing wrong complex geometries. Secondly, the structural principles deduced from SM5-1 may also affect the choice of residues to be considered in antibody optimization procedures (e.g. phage display).

### The Dom-II-SM5-1 complex can be readily superimposed without clashes onto models of entire gB

**Figure 7:**
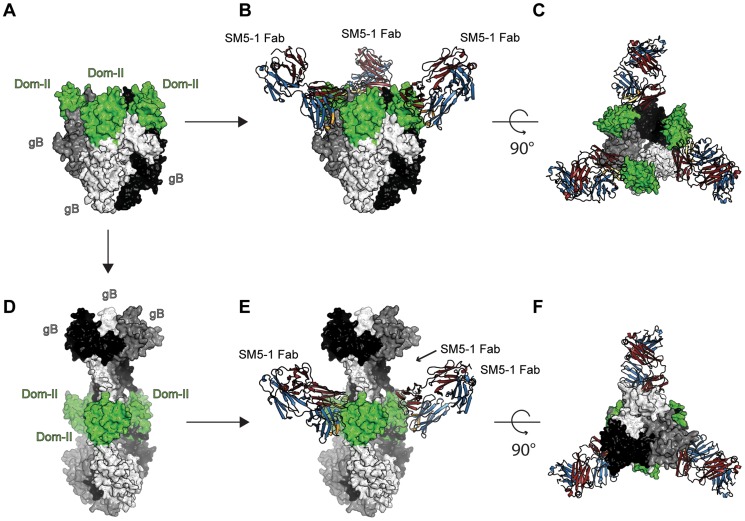
SM5-1 Fab is able to bind to both a prefusion and a postfusion model of entire trimeric HCMV gB. (A) Prefusion conformation of trimeric HCMV gB modelled according to the crystal structure of VSV-G (PDB ID 2j6j, [19], [26]). (B and C) Two orthogonal views of SM5-1 Fab bound to the prefusion conformation of gB. (D) Postfusion conformation of HCMV gB modelled according to the crystal structure of HSV-1 gB (PDB ID 2gum, [18]). (E and F) SM5-1 bound to the postfusion conformation of gB. The arrow linking panel A to C indicates the conformational transition from a prefusion to a postfusion model of HCMV gB. The arrows linking panel A and B as well as D and E represent SM5-1 binding. Trimeric gB is shown in a surface representation and colored white, grey and black with gB Dom-II highlighted in green. SM5-1 Fab is shown in a cartoon representation.

As for any viral fusion protein, HCMV gB is assumed to adopt two different conformational states, namely a pre- and postfusion conformation. Moreover, the transition from the pre- to the postfusion state is expected to facilitate the fusion of the viral envelope with the host cell membrane. However, in case of gB proteins, the exact extent of the conformational rearrangement during the transition is far from clear. Currently, crystal structures of gB proteins are only available from HSV-1 and EBV [17], [18]. These crystal structures are considered to display the postfusion conformation. Only in case of a more distantly related class III fusion protein, namely protein G from VSV, both the prefusion and postfusion conformations have been experimentally characterized [19], [20], [27]. In order to visualize, how SM5-1 could possibly recognize entire HCMV gB, we considered two gB models, namely one derived from the crystal structure of HSV-1 gB (postfusion state) and one modelled according to the prefusion conformation of VSV-G ([26] and data not shown) (Fig. 7).

In the model of HCMV gB derived from the crystal structure of HSV-1 gB, the antibody-binding epitope is fully accessible from the solvent [26]. The binding mode of SM5-1 to Dom-II can be readily transferred onto entire gB without notable steric hindrances (Fig. 7D, E and F). This also holds true if Dom-II is extended by an additional α-helix that became visible in a recent crystal structure of HSV-1 gB and that was previously absent either because of high flexibility and/or proteolytic cleavage [18], [31]. It is not clear whether this helix would actually form in HCMV since HCMV gB is physiologically cleaved *in vivo* after this segment (see above). The corresponding segment also lacks multiple hydrophobic residues that could anchor this segment to the Dom-II core structure. However, if such a helix is added to the gB-bound SM5-1 model then additional antibody antigen interactions possibly arise, namely between CDR L3 of SM5-1 and gB (Fig. S6). By contrast, L3 does not participate in Dom-II binding in the present crystal structure. Overall, the antigen-binding mode observed in SM5-1 in complex with Dom-II is fully compatible with an HCMV gB structure modelled upon the putative postfusion conformation observed in HSV-1 gB. This binding mode is further supported by recent electron microscopy studies that showed that addition of a HSV-2 Dom-II specific monoclonal antibody C226 to HSV-2 gB yields electron microscopy images that fully agree with the postfusion model of HCMV gB in complex with SM5-1 presented here [44]. Furthermore the dissociation constants of SM5-1 against isolated recombinant Dom-II and entire HCMV gB are very similar, arguing strongly against the fact that additional epitopes present on gB are missing in the Dom-II preparation [26].

This agreement also holds true if one includes potential glycosylation sites of HCMV gB in these considerations. As a mature protein, gB consists of a proteolytically processed two-chain disulfide-linked protein (residues 1–459 and 460–906), and in SDS-PAGE experiments the N-terminal part represents a diffusely migrating band indicating significant modification by glycosylation [33], [34]. Computer-based analysis for potential N-linked glycosylation sites reveals 8 sites in the N-terminal part, while the C-terminal part contains no predicted site, which is consistent with its migration as a defined band in SDS-PAGE. Adding complex N-linked glycans to the gB model still allows for unrestricted access of the antibody-binding epitope of Dom-II by SM5-1 (Fig. S7).

An HCMV gB model derived from the crystal structure of VSV-G is less reliable than an HSV-1 gB-derived model because of significant lower sequence identity between HCMV gB and VSV-G. Nevertheless, the overall structural similarity between the Dom-II segments of VSV-G and HCMV gB can be readily identified even if the respective domains superimpose only poorly (see above). When modelling HCMV gB based on the prefusion conformation of VSV-G, the Dom-II antibody-binding epitope remains accessible, and the crystal structure of Dom-II in complex with SM5-1 can again be superimposed on the VSV-G-derived HCMV gB model without any severe clashes (Fig. 7A,B and C). Thus, from these models it can be concluded that SM5-1 should be able to recognize entire gB irrespectively of whether gB adopts a conformation similar to the postfusion conformation observed in HSV-1 gB or the prefusion conformation of VSV-G.

SM5-1 and related antibodies were isolated using recombinant gB, and it has been suggested that during eukaryotic production the truncated ectodomain of gB preferentially adopts the postfusion conformation which is also remarkably stable [45], [46]. Nevertheless, the fact that SM5-1 can bind to extracellular virions as presented in conventional neutralization assays shows that SM5-1 binds also to the prefusion conformation of gB *in vivo* in agreement with the above described gB-binding models.

### Potential mode of action of SM5-1

SM5-1 neutralizes HCMV at a post-attachment state [26]. Therefore it can be excluded that SM5-1 merely blocks the attachment of the virus to the host cell. Also, the SM5-1 Fab fragment shows similar virus-neutralization capacities as complete IgG, indicating that crosslinking of gB on the viral surface is not required for neutralization (N. Spindler, unpublished results). It remains unclear, which step of the viral entry pathway is blocked upon SM5-1 binding. One possibility is, that SM5-1 prevents formation of the active fusion complex by impeding the interaction of gB with gH/gL. Congruently to our previous publications on HCMV gB, Dom-II was identified as crucial target for neutralizing antibodies in HSV gB [28], [32]. Atanasiu et al. reported for HSV-1 that anti-Dom-II antibodies were able to inhibit cell-cell fusion and block the interaction of gB with the gH/gL complex [28]. Occupation of the gH/gL-binding site in gB by SM5-1 may thus interfere with gB-gH/gL interaction. Whether a similar mode of action results in neutralization of free virions remains to be shown. Of note, in the case of EBV gB domains III, IV, and V are postulated as gH/gL interaction domains [47].

As an alternative to blocking the gH/gL-binding site, SM5-1 could also neutralize HCMV by inhibiting the transition from the prefusion to the postfusion state of gB and thereby impede membrane fusion. Although the affinity of SM5-1 for the prefusion state of gB is currently not known, circumstantial evidence as well as the above derived models suggest that SM5-1 is able to form thermodynamically stable complexes with both the prefusion and postfusion conformation of gB. However, this does not rule out that binding of SM5-1 to gB is able to kinetically inhibit the conformational transition. In the absence of data on how the fusion complex is activated on the virus envelope, the neutralizing mechanism of SM5-1 remains speculative and must await further studies.

Vaccination using recombinantly produced gB incompletely protects against HCMV infection/disease [24], [25]. The reasons for this are unknown. While the vaccine induced gB-binding and virus neutralizing antibodies comparable to natural infection when sera were analyzed in conventional fibroblast-based assays, the specific neutralizing activities were on average 15-fold lower than those observed following natural infection when epithelial cells were used as target cell type [48]. Also, the specificity of antibodies with respect to recognition of individual antigenic domains on gB has been found to differ between naturally HCMV infected and gB-vaccinated individuals [49]. This might again be directly linked to the fact that gB exists in a pre- and postfusion conformation which may differ considerably. Vaccination with gB might give rise to antibodies that recognize the postfusion state with higher affinity than the prefusion state, whereas a reversed antibody-binding preference could arise during a natural HCMV infection. Current technology allows for the comparative analysis of the individual antibody repertoire following infection and vaccination and this may provide valuable information for the future optimization of gB-based vaccines.

Obviously, further investigations are needed to determine how exactly SM5-1 blocks the viral entry mechanism. Clearly, the crystal structure presented here will be valuable for further exploring the prefusion and postfusion state of HCMV. However, more importantly, the structural insights provided here open up a window of opportunity for the structure-based design of HCMV vaccines.

## Materials and Methods

### Production and purification of recombinant Dom-II Proteins

For the expression of GST fusion proteins of engineered variants of Dom-II in which residues 112 to 132 and 344 to 438 of the HCMV gB protein were linked by a 5-residue long artificial linker sequence, the respective coding sequences were cloned into pGEX-6P-1 plasmids (GE Healthcare) as described and DH10B bacteria were transformed with the expression vectors [32]. The fusion proteins were purified from *Escherichia coli* lysates following incubation with glutathione Sepharose 4B (GE Healthcare) for 2 h at room temperature. Unbound bacterial protein was removed by washing with PBS, and proteins were eluted with 10 mM reduced L-glutathione. To remove the GST part, the bound Dom-II fusion protein was proteolytically cleaved using PreScission protease (GE Healthcare) by incubation for 5 h at 4°C and the Dom-II part was eluted. For further purification anion-exchange chromatography in 20 mM Tris, pH 8.0 was performed using a HiTrapQ FF 1-ml column (GE Healthcare) on an ÄKTApurifier (GE Healthcare) followed by size exclusion chromatography in 20 mM Tris, 150 mM NaCl, pH 7.4 on a HiLoad 16/60 Superdex75 column (GE Healthcare).

### Preparation of SM5-1 Fab fragment and purification of the antigen-antibody-complex

The Fab fragment was prepared by papain digestion of the SM5-1 IgG molecule using a Fab preparation Kit from Thermo Scientific Pierce (Germany). Papain digestion was carried out according to the manufacturer's instructions. The Fc-part and undigested IgG molecules were removed by affinity purification using a HiTrap Protein A HP 1-ml column (GE Healthcare). Size exclusion chromatography in 20 mM Tris, 150 mM NaCl, pH 7.4 was used as final purification step of the Fab fragment using a HiLoad 16/60 Superdex200 column. For complex formation, purified SM5-1 Fab and Dom-II were co-incubated at a ratio of 1∶2 (*w/w*) followed by size exclusion chromatography in 20 mM Tris, 150 mM NaCl, pH 7.4 using a HiLoad 16/60 Superdex200 column to remove non-complexed proteins.

### Crystallization and structure determination

Engineered Dom-II was crystallized using the hanging-drop vapor-diffusion method and equilibrating a 1–4 µl mixture containing protein solution (11.9 mg/ml Dom-II in 20 mM Tris-HCl, pH 7.5) and reservoir solution (1.5 M Li_2_SO_4_, 0.1 M Na-HEPES salt, pH 7.5) at ratios 1∶2.5 or 1∶3 against 700 µl of reservoir solution. Crystallization of the SM5-1 Fab fragment was achieved with the sitting-drop vapor-diffusion set-up. 200 nl of protein solution (9.7 mg/ml in 20 mM Tris-HCl, pH 7.5) were mixed with 200 nl of reservoir solution (4 M sodium formate) and equilibrated against reservoir solution. Crystals of individual Dom-II and SM5-1 Fab grew at 19°C within two days. In both cases, crystals were flash-cooled in liquid nitrogen without addition of cryoprotectants.

Dom-II of gB in complex with SM5-1 was crystallized with the sitting-drop vapor-diffusion method using protein concentrations between 10 and 15 mg/ml in 20 mM Tris-HCl, pH 7.5 and mixing 200 nl of protein solution with 200 nl of reservoir solutions. To increase the chances for obtaining well-diffracting crystals of the complex, we crystallized SM5-1 in complex with several different mutants of Dom-II that bind SM5-1 with similar affinities than wild-type Dom-II [32]. However, most complexes only produced crystals that were intergrown and unsuitable for X-ray diffraction experiments. In case of the complex formed between double mutant gB Dom-II-I397A/N398A and SM5-1 Fab, single crystals could be isolated after equilibrating the protein-reservoir mixture against 70 µl reservoir solution (0.1 M HEPES, pH 7.5, 0.2 M L-proline, and 24% PEG 1500) for one month at 19°C. Crystals of the complex were briefly soaked with 20% ethylene glycol before being transferred into liquid nitrogen. In the crystal structure, and as expected, the residues at positions 397 and 398 in Dom-II do not participate in the interaction with SM5-1 Fab (Fig. 3).

All diffraction data sets were collected using 0.5 to 0.6° rotation frames at PX beamline BL 14.1 at Hemholtz Zentrum Berlin BESSY synchrotron facility and processed with program XDS [50]. All three crystal structures could be solved by molecular replacement using program PHASER within the CCP4 program suite [51], [52]. The structure of Dom-II comprising residues 118 to 132 and 344 to 438 of the HCMV gB protein could be solved with a mixed search model that was based on the corresponding Dom-II from HSV-1 glycoprotein B (residues 142 to 152 and 364 to 459, PDB ID 2gum). The mixed model was generated using the Joint Center of Structural Genomics SCRWL server [53], [54].

The unrelated Fab fragment of the human anti-HIV-1 gp120 reactive antibody E51 (PDB ID 1rzf) was used as a search model for the SM5-1 Fab structure. Flexible parts, such as CDR loops were deleted and both variable domains and constant domains were considered separately during the molecular replacement calculations. Ensemble 1: residues 109 to 210 (CL) and 114 to 214 (CH1); ensemble 2: residues 2–23, 34–54, 63–91, 96–106 (VL) and 2–23, 33–51, 57–95, 103–111 (VH). The gB Dom-II-SM5-1 Fab complex structure was solved using the crystal structures of the previously solved individual proteins as search models. Here also, any flexible regions in SM5-1 were removed and two different ensembles consisting of either the variable or constant domains used during the molecular replacement search of the SM5-1 fragment. All structures were refined with the CCP4 program REFMAC5 until convergence and until no further details could be interpreted in the electron density maps [52], [55]. During the final refinement rounds a TLS refinement step was performed in order to improve the fit between the models and the experimental data.

The elbow angle of the SM5-1 Fab fragment was calculated with the AS2TS web server using the following immunoglobulin domain boundaries: V_L_: residues 1 to 110; V_H_: 1 to 131, C_L_: 111 to 212 and C_H1_: 132 to 232 [36], [56]. Changes in the accessible surface areas were calculated with program AREAIMOL from the CCP4 program suite using the default solvent probe radius of 1.4 Å [52]. All molecular illustrations were generated with program PyMol (http://www.pymol.org/citing). Potential N-linked glycosylation sites were predicted using the NetNGlyc server [57].

### Expression of the SM5-1*germ* variant and virus neutralization

The coding sequences of SM5-1 and a partially germline-reverted mutant derivative (SM5-1*gern*) were constructed according to Fig. S3. The respective nucleotide sequences coding for the heavy and light chain variable regions were chemically synthesized by Life Technologies (Germany) and cloned into eukaryotic IgG expression vectors [58]. To produce IgG, 293T cells were co-transfected with respective IgG heavy and light chain expression vector plasmids. The cell culture supernatant was harvested 8 days post transfection and IgG was purified using a HiTrap Protein A HP 1-ml column (GE Healthcare) on an ÄKTAprime chromatography system (GE Healthcare). Virus infection of fibroblasts and neutralization was exactly as described in Pötzsch et al. 2011 [26].

### MD simulations

Molecular dynamics (MD) simulations were performed for SM5-1 and for a 6-fold *in silico* variant (SM5-1*), in which the respective residues were exchanged to match the sequence of the less mature SM1-6 (K30T, D31G, H32Y, H115Y, N116D and R117V). For both SM5-1 and SM5-1* two independent simulations were performed starting either from the conformation present in complex with Dom-II or from the crystal structure of unbound SM5-1. This resulted in a total of four simulations. To reduce computational cost, only the V_H_-V_L_ domain (aa 1–131 and 1–110 of chain H and L, respectively) of the Fab-fragment were included in the simulation. The protonation state of titratable amino acids was calculated by PROPKA [59] and the Swiss PDB Viewer was applied to add unresolved residues and side chains, as well as for the amino acid exchanges [60].

All MD simulations were performed using the AMBER 11 program package and the ff99SB force field parameters [61], [62]. Proteins were placed in periodic truncated octahedral water boxes with at least 15 Å of solvent between any atom of the solute and the periodic box edges. The systems were neutralized by adding the respective number of Cl^−^ ions. Initially, all four systems were minimized in a three-step procedure and then gradually heated to 310 K following a previously established protocol [63], [64]. Subsequently, 100 ns MD simulations were performed with a time step of 2 fs and periodic boundaries. The Settle-algorithm [65] was applied to constrain covalent bonds involving hydrogens. The obtained data of the simulation was analyzed and visualized using AMBER Tools and VMD [66].

### Accession numbers

Coordinates and structure factors of Dom-II, SM5-1 Fab, and the Dom-II-SM5-1 Fab complex have been deposited with the Protein Data Bank (PDB) under accession codes 4OSN, 4OSU, and 4OT1, respectively.

## Supporting Information

Figure S1 **Accessible surface areas buried per residue in the interface between Dom-II and SM5-1.** Changes in accessible surface area for (A) Dom-II residues, (B) SM5-1 light chain residues and (C) heavy chain residues upon complex formation. The artificial linker residues in Dom-II are marked by a dashed line and the mutations I397A and N398A present in Dom-II used for the complex formation are marked by asterisks.
(TIF)

Figure S2 **Details of the interaction between SM5-1 and Dom-II.** (A) CDR H3 residue Leu109 does not completely fill a hydrophobic pocket located on the surface of Dom-II. The pocket was calculated with the program VOIDOO [68]. (B) Contacts mediated by the Dom-II (green) YK epitope. Mutational analysis identified Tyr364 and Lys379 as the main determinants for high affinity SM5-1 binding. Lys379 together with Glu359 is the only residue that contacts both CDRs L1 (yellow) and H3 (orange). Tyr364 possibly plays a role in positioning Lys379 and Glu359 without directly contacting SM5-1.
(TIF)

Figure S3 **Sequence alignment of relevant portions of SM5-1 and of partially germline-reverted SM5-1** ***germ*** **.** In SM5-1*germ* all CDRs with the exception of CDRs L1 and H3 were reverted to the germline sequence. In SM5-1*germ* amino acids were mutated as indicated. * indicate identical sequence.
(TIF)

Figure S4 **Polar interactions and hydrogen bonds formed at the base of CDR H3.** MD simulations show that the removal of these interactions increases the flexibility of CDR H3. We propose that these residues contribute to the higher affinity of antibody SM5-1 in comparison to less affinity-maturated antibodies.
(TIF)

Figure S5 Conformational flexibility of SM5-1 and SM5-1* using the crystal structure of free SM5-1 as starting conformation. (A) Plot of the root mean square fluctuations (RMSF) per residue indicating the enhanced flexibility of residues 102–112 (CDR H3) in SM5-1* (red line) compared to SM5-1 (black line). Sequence positions that differ between SM5-1 and SM5-1* are marked by a yellow asterisk. (B, C) Overlay of 6 structures collected every 20 ns over the simulation time for SM5-1 (B) and SM5-1* (C). Note that the CDR H3 loop in SM5-1* exhibits a higher flexibility and deviates further from the starting structure. The six residues that are different between SM5-1 and SM5-1* are shown in stick presentation and a pink arrow points towards the CDR H3 loop.
(TIF)

Figure S6 **Model showing a putative interaction of SM5-1 with a C-terminally extended Dom-II domain.** Recently an additional α-helix (red) became visible in the crystal structure of the low-pH form of HSV-1 gB (PDB ID 3nwf, [31]). Although previously overlooked, it is possible that this C-terminal helical segment is an integral part of gB Dom-II domains. However this segment (HCMV gB residues 443–455) was not present in engineered HCMV Dom-II used for the crystal structure determination of the Dom-II-SM5-1 complex. If modelled into the crystal structure, then it appears that (i) the helix does not interfere with the architecture of the complex and (ii) the helix possibly generates additional contacts between Dom-II and SM5-1, in particular CDR L3.
(TIF)

Figure S7 **Model for the neutralization of glycosylated HCMV gB by SM5-1.** (A) SM5-1 bound to Dom-II in a trimeric postfusion model of gB [26]. (B) Model of glycosylated HCMV generated using the GlyProt web server [69]. (C) Model showing that SM5-1 can access the Dom-II-binding site even if in case that gB is glycosylated. (D) In SM5-1 bound to gB the C-termini of two neighboring SM5-1 Fab C_H_ domains are positioned more than 150 Å apart from each other. Therefore, two Fab segments from a single IgG molecule will not be able to bind simultaneously to the same gB trimer. Hence, IgGs very likely crosslink different gB trimers on the HCMV surface. The crystal structure of HSV-1 gB (PDB ID 2gum, [18]) was used as a template for the modelling of HCMV gB. In each panel trimeric gB is shown in white, grey and black, respectively, with the Dom-II segment highlighted in green. SM5-1 is displayed in a cartoon representation and sugar atoms as spheres.
(TIF)

Table S1 **Binding affinity of various SM antibodies for gB and their respective neutralization activity.**
(DOCX)
