# Supplementary figures and images for: Structural Basis for the Recognition of Human Cytomegalovirus Glycoprotein B by a Neutralizing Human Antibody
Source: PLoS Pathog. 2014 Oct 9;10(10):e1004377. doi: 10.1371/journal.ppat.1004377 (PMC4192593; doi:10.1371/journal.ppat.1004377)

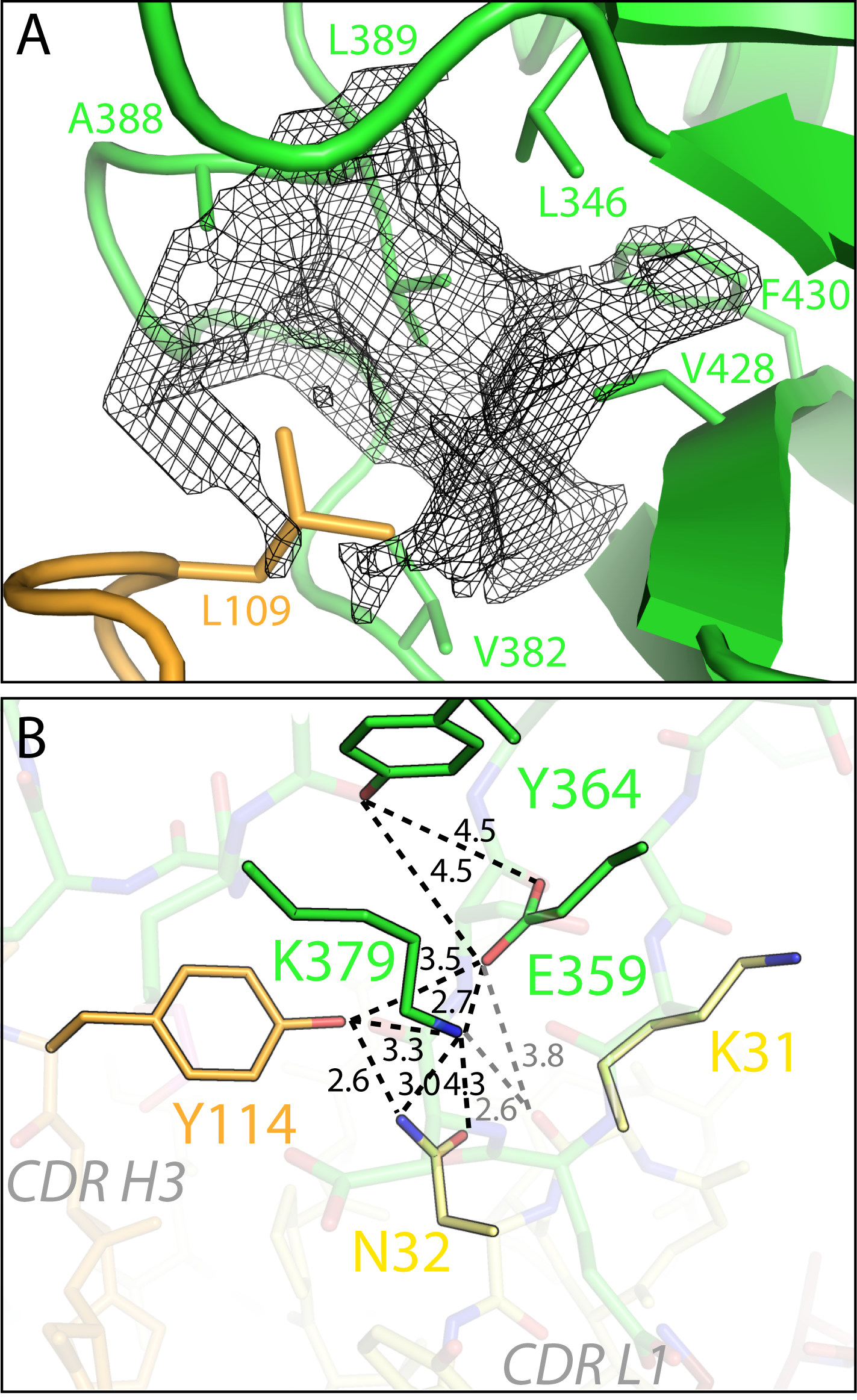

Supplement: Figure S2 — Details of the interaction between SM5-1 and Dom-II. (A) CDR H3 residue Leu109 does not completely fill a hydrophobic pocket located on the surface of Dom-II. The pocket was calculated with the program VOIDOO [68]. (B) Contacts mediated by the Dom-II (green) YK epitope. Mutational analysis identified Tyr364 and Lys379 as the main determinants for high affinity SM5-1 binding. Lys379 together with Glu359 is the only residue that contacts both CDRs L1 (yellow) and H3 (orange). Tyr364 possibly plays a role in positioning Lys379 and Glu359 without directly contacting SM5-1. (TIF) [file ppat.1004377.s002.tif]

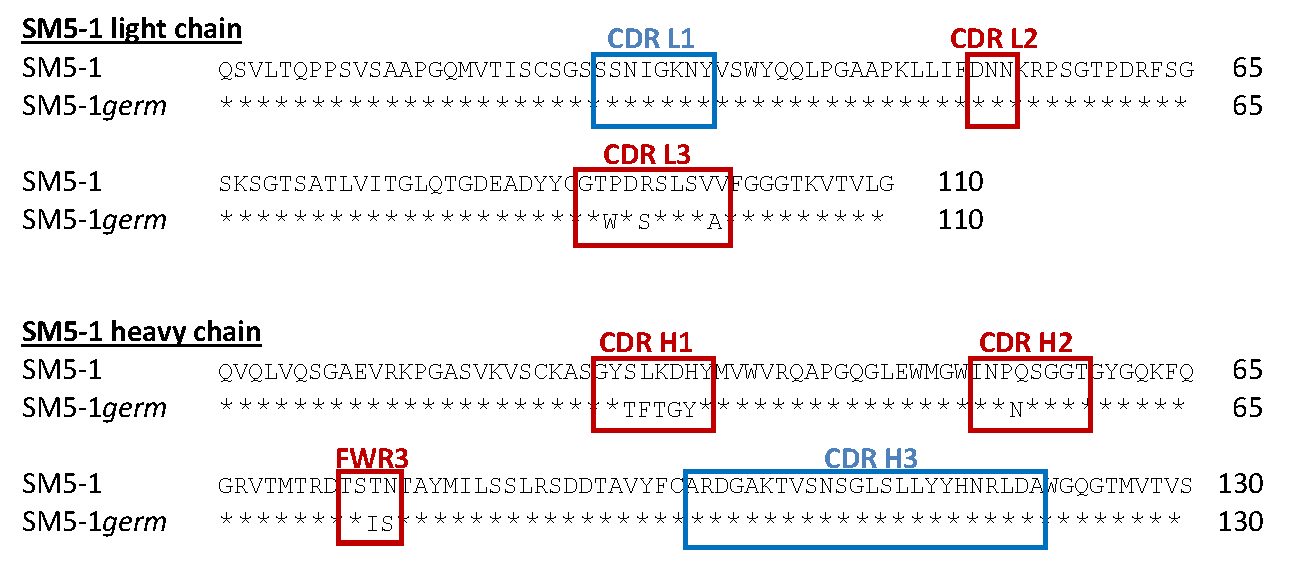

Supplement: Figure S3 — Sequence alignment of relevant portions of SM5-1 and of partially germline-reverted SM5-1 germ . In SM5-1germ all CDRs with the exception of CDRs L1 and H3 were reverted to the germline sequence. In SM5-1germ amino acids were mutated as indicated. * indicate identical sequence. (TIF) [file ppat.1004377.s003.tif]

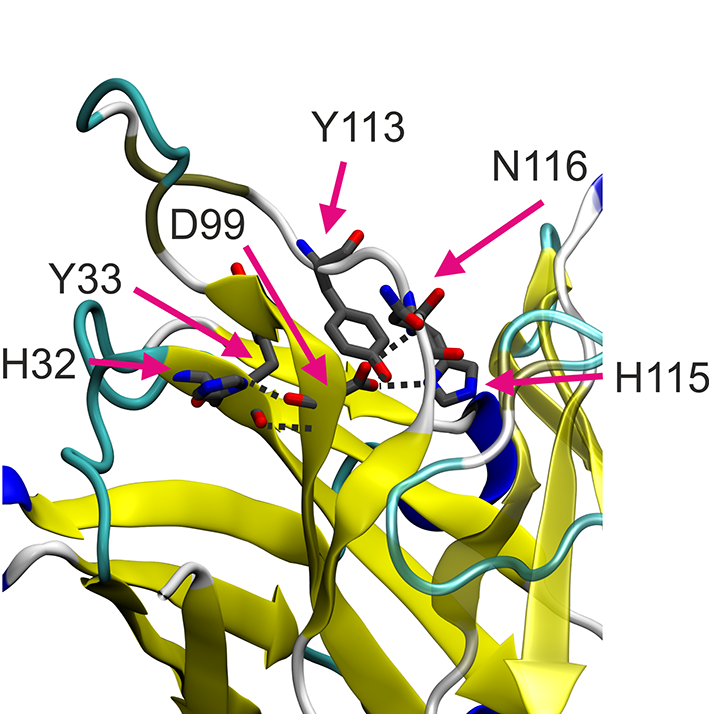

Supplement: Figure S4 — Polar interactions and hydrogen bonds formed at the base of CDR H3. MD simulations show that the removal of these interactions increases the flexibility of CDR H3. We propose that these residues contribute to the higher affinity of antibody SM5-1 in comparison to less affinity-maturated antibodies. (TIF) [file ppat.1004377.s004.tif]

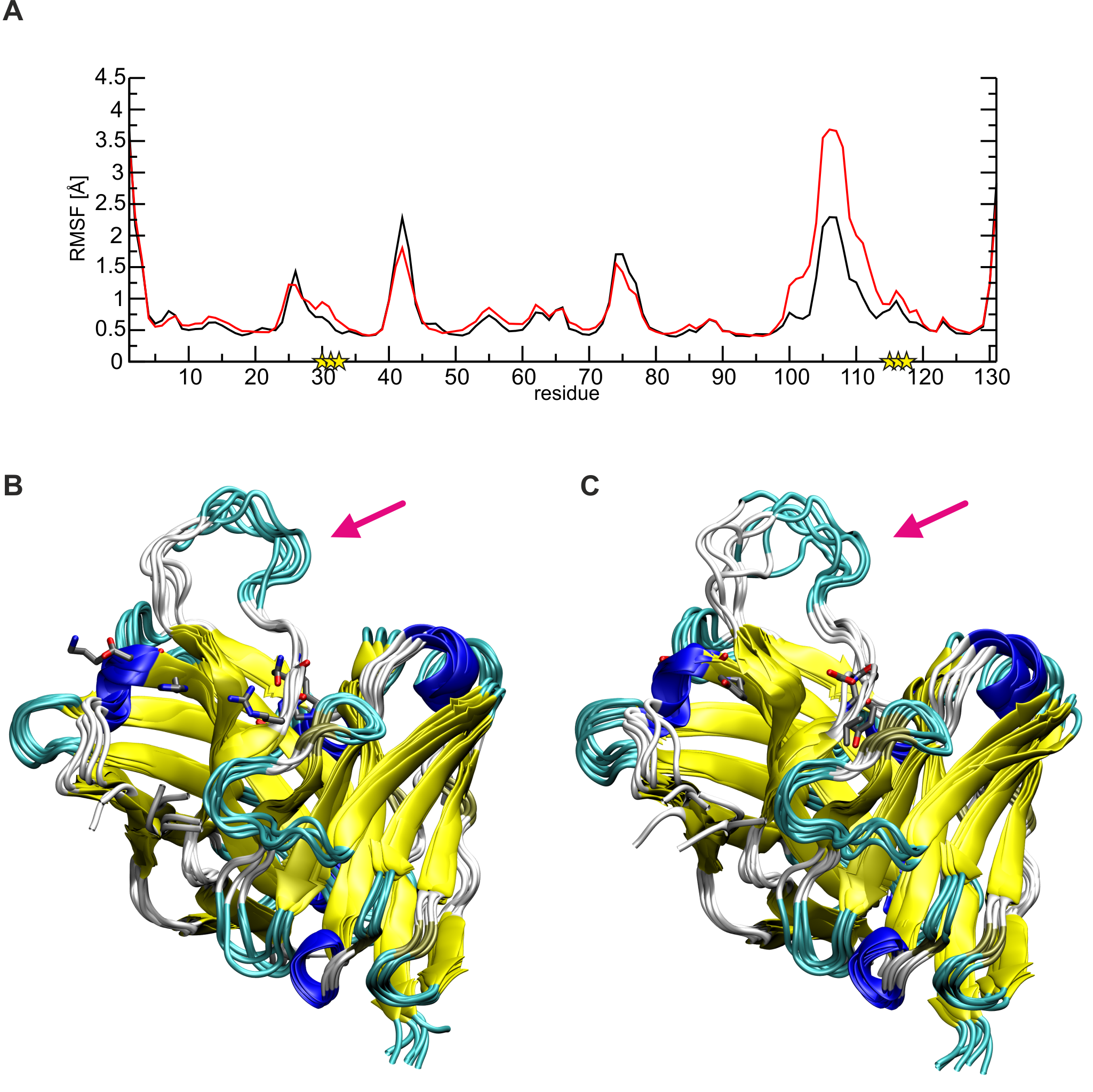

Supplement: Figure S5 — Conformational flexibility of SM5-1 and SM5-1* using the crystal structure of free SM5-1 as starting conformation. (A) Plot of the root mean square fluctuations (RMSF) per residue indicating the enhanced flexibility of residues 102–112 (CDR H3) in SM5-1* (red line) compared to SM5-1 (black line). Sequence positions that differ between SM5-1 and SM5-1* are marked by a yellow asterisk. (B, C) Overlay of 6 structures collected every 20 ns over the simulation time for SM5-1 (B) and SM5-1* (C). Note that the CDR H3 loop in SM5-1* exhibits a higher flexibility and deviates further from the starting structure. The six residues that are different between SM5-1 and SM5-1* are shown in stick presentation and a pink arrow points towards the CDR H3 loop. (TIF) [file ppat.1004377.s005.tif]

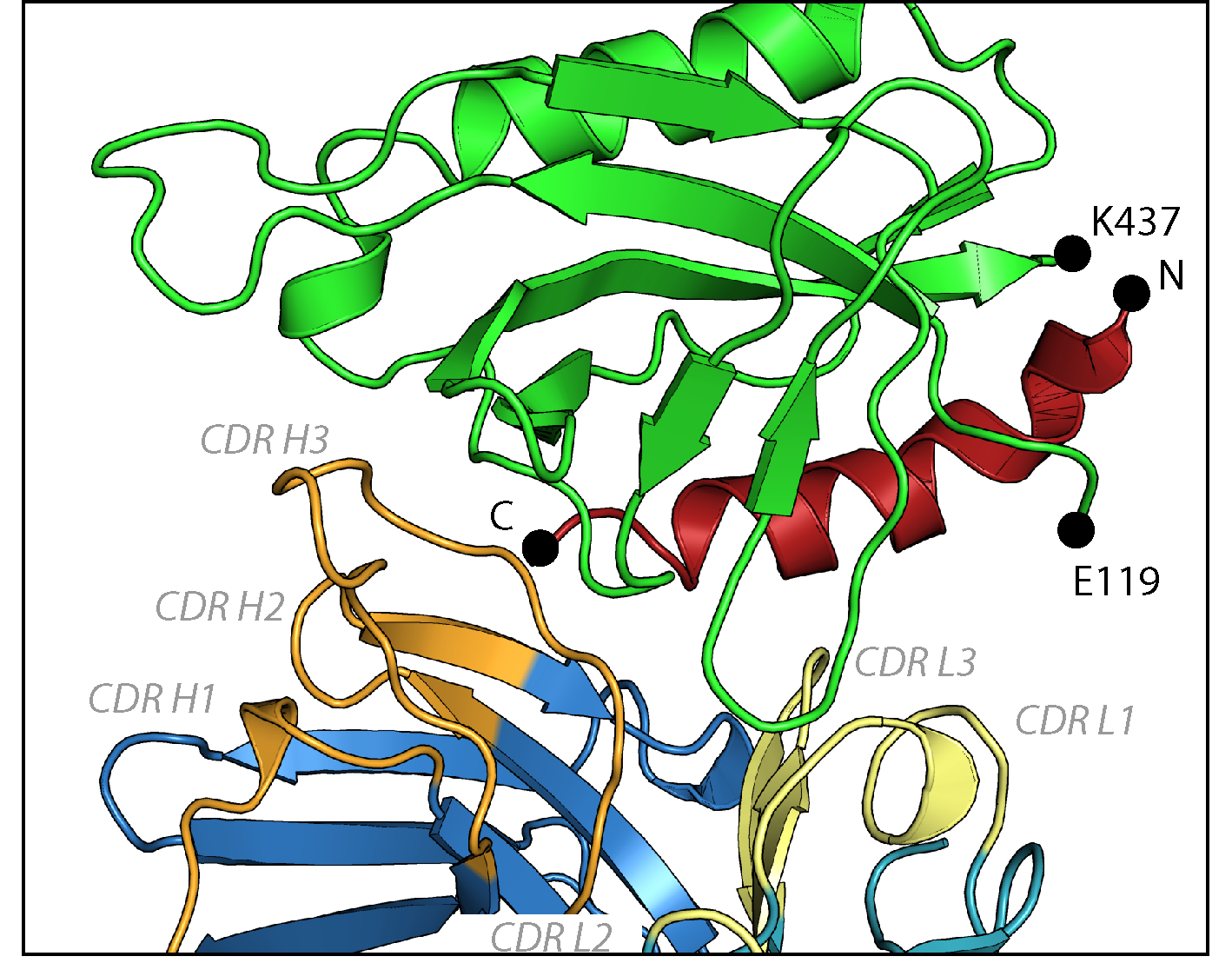

Supplement: Figure S6 — Model showing a putative interaction of SM5-1 with a C-terminally extended Dom-II domain. Recently an additional α-helix (red) became visible in the crystal structure of the low-pH form of HSV-1 gB (PDB ID 3nwf, [31]). Although previously overlooked, it is possible that this C-terminal helical segment is an integral part of gB Dom-II domains. However this segment (HCMV gB residues 443–455) was not present in engineered HCMV Dom-II used for the crystal structure determination of the Dom-II-SM5-1 complex. If modelled into the crystal structure, then it appears that (i) the helix does not interfere with the architecture of the complex and (ii) the helix possibly generates additional contacts between Dom-II and SM5-1, in particular CDR L3. (TIF) [file ppat.1004377.s006.tif]

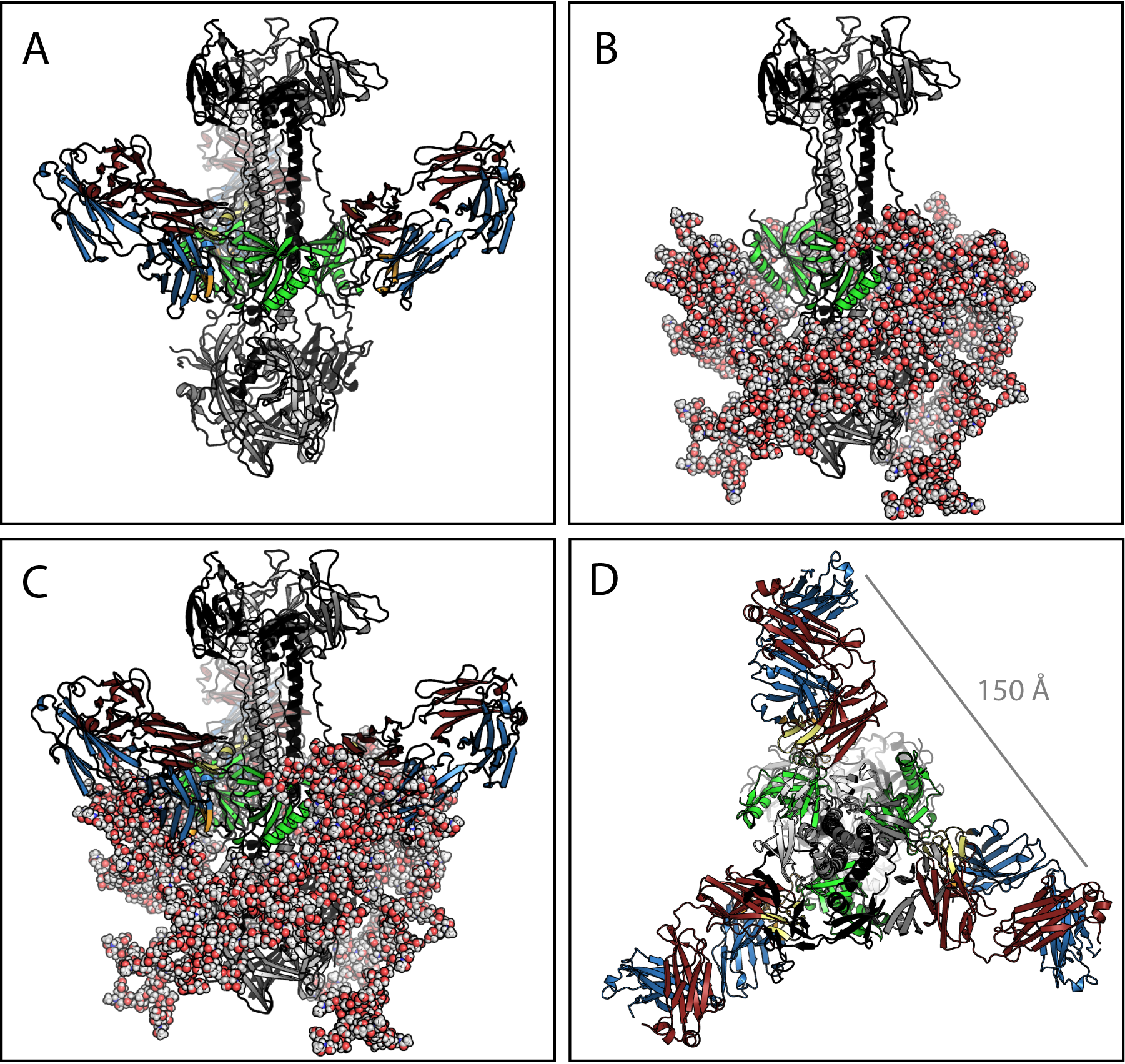

Supplement: Figure S7 — Model for the neutralization of glycosylated HCMV gB by SM5-1. (A) SM5-1 bound to Dom-II in a trimeric postfusion model of gB [26]. (B) Model of glycosylated HCMV generated using the GlyProt web server [69]. (C) Model showing that SM5-1 can access the Dom-II-binding site even if in case that gB is glycosylated. (D) In SM5-1 bound to gB the C-termini of two neighboring SM5-1 Fab CH domains are positioned more than 150 Å apart from each other. Therefore, two Fab segments from a single IgG molecule will not be able to bind simultaneously to the same gB trimer. Hence, IgGs very likely crosslink different gB trimers on the HCMV surface. The crystal structure of HSV-1 gB (PDB ID 2gum, [18]) was used as a template for the modelling of HCMV gB. In each panel trimeric gB is shown in white, grey and black, respectively, with the Dom-II segment highlighted in green. SM5-1 is displayed in a cartoon representation and sugar atoms as spheres. (TIF) [file ppat.1004377.s007.tif]
