# Supplementary material for: Structural Basis for the Recognition of Human Cytomegalovirus Glycoprotein B by a Neutralizing Human Antibody
Source: PLoS Pathog. 2014 Oct 9;10(10):e1004377. doi: 10.1371/journal.ppat.1004377 (PMC4192593; doi:10.1371/journal.ppat.1004377)
Supplement: Table S1 — Binding affinity of various SM antibodies for gB and their respective neutralization activity. (DOCX) [file ppat.1004377.s008.docx]

**Table S1. Binding affinity of various SM antibodies for gB and their respective neutralization activity**

|  | k_a_ (1/Ms)* | k_d_ (1/s) | K_D_ (M) | 50% neutralization activity  (µg/ml)** |
| --- | --- | --- | --- | --- |
| SM1-6 | 7.6 x 10^4^ | 5.1 x 10^-4^ | 6.8 x 10^-9^ | 1.3 |
| SM3-1 | 5.6 x 10^4^ | 8.4 x 10^-4^ | 1.5 x 10^-8^ | 1.2 |
| SM6-5 | 7.7 x 10^4^ | 1.2 x 10^-4^ | 1.5 x 10^-9^ | 0.5 |
| SM5-1 | 4.0 x 10^5^ | 2.4 x 10^-5^ | 5.7 x 10^-11^ | 0.3 |
| SM4-3 | 4.1 x 10^5^ | 1.5 x 10^-4^ | 3.6 x 10^-10^ | 0.6 |
| SM11-17 | 5.7 x 10^4^ | 3.2 x 10^-4^ | 5.6 x 10^-9^ | 1.0 |

* All SM antibodies were isolated and analyzed by surface plasmon resonance (SPR) as previously described ([1](#_ENREF_1)). K_D_ was calculated as follows: K_D_ = k_d_/k_a_

** Please note that these neutralization activities have already been reported in our previous publication ([1](#_ENREF_1)).

1. Potzsch, S., Spindler, N., Wiegers, A.K., Fisch, T., Rucker, P., Sticht, H., Grieb, N., Baroti, T., Weisel, F., Stamminger, T. *et al.* (2011) B cell repertoire analysis identifies new antigenic domains on glycoprotein B of human cytomegalovirus which are target of neutralizing antibodies. *PLoS pathogens*, **7**, e1002172.
